# Multiparametric quantitative MRI combining SyMRI and MUSE-DWI for noninvasive stratification of HER2 status in breast cancer

**DOI:** 10.3389/fonc.2025.1709170

**Published:** 2025-12-05

**Authors:** Kui Yang, Wei Zhang, Hao Zheng, Da Shuang Ji, Hu Chang, Feng Li

**Affiliations:** 1 Department of Radiology, Xiangyang Central Hospital, Affiliated Hospital of Hubei University of Arts and Science, Xiangyang, Hubei, China; 2 School of Psychology, Shandong Second Medical University, Weifang, Shandong, China

**Keywords:** breast cancer, HER2, HER2-low, synthetic MRI (SyMRI), MUSE-DWI, multiparametric MRI, noninvasive stratification

## Abstract

**Background:**

Accurate stratification of HER2 status is crucial for treatment decision-making and prognostic evaluation in breast cancer. With the recognition of HER2-low as a distinct subtype, which has recently gained clinical relevance as HER2-low patients may benefit from emerging HER2-targeted therapies, conventional pathological methods remain the gold standard; however, they are invasive and prone to sampling bias, and may not fully reflect intratumoral heterogeneity. Imaging provides a noninvasive alternative for evaluating HER2 expression. This study aimed to assess the value of synthetic MRI (SyMRI) combined with multiplexed sensitivity encoding diffusion-weighted imaging (MUSE-DWI) for noninvasive stratification of HER2 status in breast cancer.

**Methods:**

A total of 138 patients with pathologically confirmed invasive breast cancer underwent standardized MRI protocols, including SyMRI, MUSE-DWI, and DCE-MRI before biopsy or any treatment. Quantitative parameters (T_1_, T_2_, PD, ADC, and their pre-/post-contrast changes) were measured. Differences among HER2-zero, HER2-low, and HER2-overexpressing groups were analyzed. Univariate and multivariate logistic regression analyses were performed to identify independent predictors and construct nomogram models for predicting HER2 positivity and HER2-low status. Model performance was evaluated using ROC curves and calibration analysis.

**Results:**

HER2-overexpressing tumors more frequently demonstrated heterogeneous enhancement, washout-type time–intensity curves (TICs), and larger maximum diameters. In multivariable analysis, ADC, maximum diameter, T2-pre, and enhancement pattern were independent predictors of HER2 positivity (AUC = 0.940; bootstrap-corrected AUC = 0.930), whereas ADC and PD-Δ% independently predicted HER2-low status (AUC = 0.810; bootstrap-corrected AUC = 0.830). Both models showed good discrimination and calibration, and decision curve analysis indicated a favorable net clinical benefit across a wide range of threshold probabilities.

**Conclusions:**

SyMRI combined with MUSE-DWI enables noninvasive stratification of HER2 status in breast cancer. The proposed models demonstrated high diagnostic performance, good calibration, and favorable clinical utility in decision curve analysis, particularly for identifying HER2-low tumors. This imaging approach has the potential to complement biopsy and assist personalized treatment planning.

## 1 Introduction

Breast cancer (BC) is the most common malignancy in women worldwide, and its incidence continues to rise, particularly among middle-aged and elderly women (1). Recent studies have also revealed that family, socioeconomic, and lifestyle factors contribute to BC risk and prevention strategies (2). Molecular subtyping plays a pivotal role in treatment selection and prognosis, among which human epidermal growth factor receptor 2 (HER2) status is of particular importance (3). HER2 overexpression (HER2-oe) is associated with aggressive tumor biology, high metastatic potential, and sensitivity to anti-HER2 therapy, whereas HER2-negative tumors lack such targeted treatment options (4).

With the development of antibody–drug conjugates (ADCs), HER2-low has been recognized as a distinct subtype (5). This subtype, defined as IHC 1+ or 2+ with negative FISH, lies between HER2-oe and HER2-zero (IHC 0) and accounts for nearly 50% of all BC cases (6). Accurate stratification of HER2-zero, HER2-low, and HER2-oe has therefore become essential for individualized therapy.

Currently, HER2 status assessment relies on IHC and FISH; however, these methods are invasive, subject to sampling bias, and unable to capture whole-tumor heterogeneity or allow repeated assessment (7). MRI provides a noninvasive and repeatable approach for tumor characterization. Diffusion-weighted imaging (DWI) reflects cellularity via ADC values but conventional SS-EPI-DWI is susceptible to distortion and artifacts (8). MUSE-DWI improves spatial resolution and reduces distortion through multi-shot acquisition and parallel imaging (9, 10). SyMRI enables simultaneous acquisition of T1, T2, and proton density (PD) maps, offering quantitative microstructural information in a single scan (11). Although SyMRI has shown value in evaluating solid tumors, its capability to differentiate HER2 expression subtypes—especially distinguishing HER2-low from HER2-zero—remains unclear (12, 13).

Therefore, this study aimed to evaluate the diagnostic performance of multiparametric MRI combining SyMRI and MUSE-DWI for noninvasive stratification of HER2 status in breast cancer. We compared imaging parameters among HER2-zero, HER2-low, and HER2-oe groups, assessed their discriminatory ability in HER2-positive versus HER2-negative classification, and identified key markers for HER2-low identification. Based on multivariate analysis, a nomogram model was developed to assist personalized clinical decision-making.

This retrospective study reviewed patients who underwent breast MRI examinations at Xiangyang Central Hospital between April 2023 and September 2024, with a total of 213 cases initially screened.

Inclusion criteria were as follows:

1. Adult female patients who were neither pregnant nor lactating, to avoid biopsy- or therapy-related tissue changes that could affect imaging results.
2. Pathologically confirmed breast cancer.
3. No prior clinical intervention or treatment before MRI examination.
4. MRI performed before any biopsy, surgery, or treatment (treatment-naïve).
5. Availability of breast DCE-MRI, SyMRI, and MUSE-DWI sequences; SyMRI scans were acquired both before contrast administration and after completion of the DCE-MRI examination, using identical acquisition parameters.

Exclusion criteria were as follows:

1. Poor image quality with severe artifacts precluding lesion characterization.
2. Non-mass enhancement pattern on contrast-enhanced MRI, because lesion margins are often indistinct and ROI placement is not reproducible.
3. Maximum lesion diameter <10 mm.

**Figure 1:**
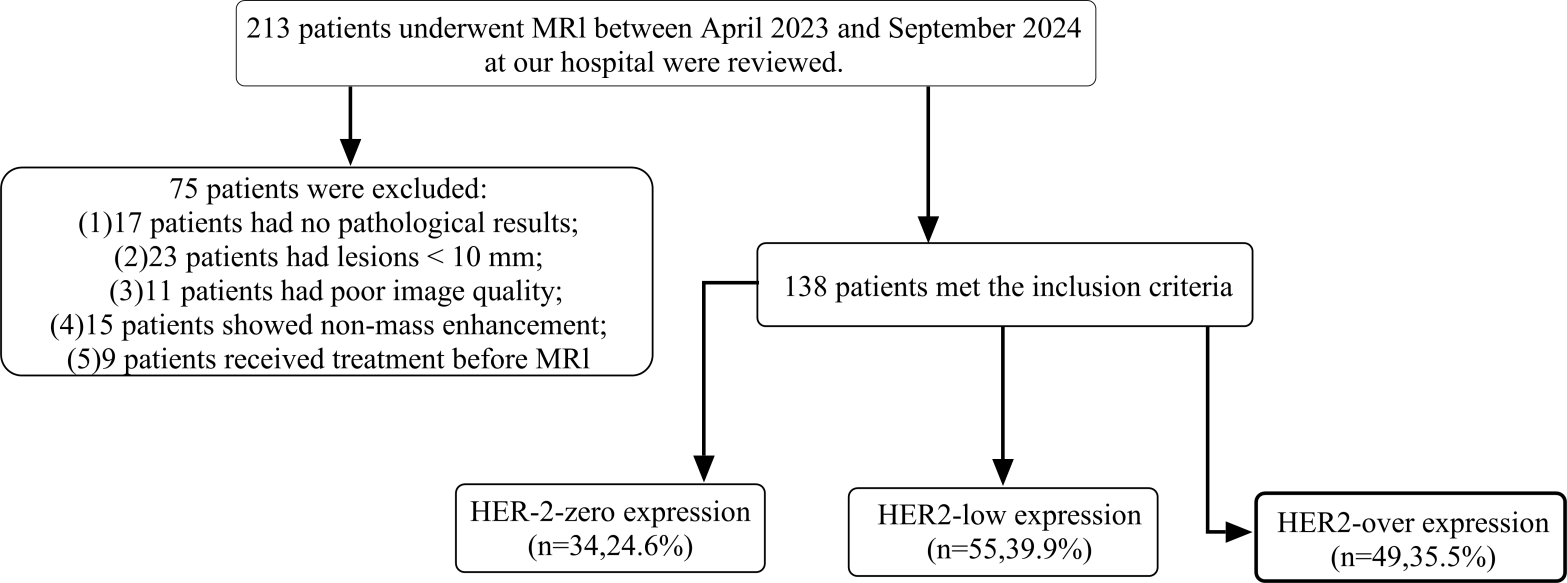
Flowchart of patient selection. A total of 213 patients who underwent breast MRI between April 2023 and September 2024 were reviewed. After applying exclusion criteria, 138 patients were included and categorized into three groups according to HER2 status: HER2-zero (n = 34), HER2-low (n = 55), and HER2-overexpression (n = 49).

Finally, 138 patients met the eligibility criteria and were included in the study. The detailed selection process is illustrated in **Figure 1**.

### 1.1 MRI examination

All patients underwent breast MRI on a 3.0-T scanner (SIGNA Architect, GE Healthcare, Milwaukee, WI, USA) in the prone, head-first position, with both breasts naturally suspended in a dedicated 16-channel phased-array breast coil. Importantly, none of the patients received any form of intervention or treatment—including core-needle biopsy, surgery, chemotherapy, radiotherapy, endocrine therapy, or anti-HER2 therapy—prior to MRI. Biopsy and surgical excision were completed within 15 days after MRI.

The imaging protocol included the following steps:

1. Conventional sequences (T1-weighted imaging [T1WI] and T2-weighted imaging [T2WI]) were first acquired. 2. An initial SyMRI sequence (MAGnetic resonance image Compilation, MAGiC) was performed, to obtain baseline T1, T2, and PD values before contrast injection. 3. Dynamic contrast-enhanced MRI (DCE-MRI) was subsequently acquired (31 phases in total). 4. A second SyMRI scan was performed immediately after DCE-MRI (≈4 min 07 s), using the same acquisition parameters as the pre-contrast SyMRI, and lasted about 4 minutes to evaluate contrast-induced changes under identical conditions. Contrast agent administration was performed via antecubital vein injection of gadoterate meglumine (Gadoteric acid injection, Hengrui Medicine, approval no. H20153167, China) at a dose of 0.2 mL/kg and an injection rate of 2 mL/s.

Detailed MRI acquisition parameters are summarized in **Table 1**.

**Table 1:** MRI acquisition parameters.

Parameter	T1WI	T2WI	DWI	SyMRI	DCE-MRI
Sequence	FSE	FLEX	MUSE-DWI	MAGiC	DISCO+C
TR(ms)	709	4258	4885	4000	5.6
TE (ms)	7.5	58	81.9	21.3,85.3	1.1
FOV(mm^2^)	340×340	340×340	340×340	250×250	340×340
Slice thickness	4	4	4	3	2.4
Number of slices	30	30	28	25	60
Matrix	325×288	325×288	128×160	512×512	288×224
B-value(s/mm^-2^)	–	–	800/1000	–	–
Acceleration factor	2	2	1	2	1
Scan time(min)	01:14	02:08	04:24	05:10	04:07

SyMRI, synthetic MRI; MAGiC, GE implementation of SyMRI; DISCO+C, time-resolved DCE-MRI.

### 1.2 Image analysis and measurements

Image analysis was independently performed by two radiologists (≥6 years of experience), blinded to pathological results. Raw SyMRI data were processed on a GE workstation (GE Healthcare, version 100.3 Ext.8) to generate T_1_-, T_2_-, and PD-maps. Based on lesion extent identified on DCE-MRI, a region of interest (ROI) was manually delineated to encompass the entire lesion while avoiding necrotic, cystic, and hemorrhagic areas. This ROI was drawn on the largest axial slice rather than on the whole tumor volume, to ensure reproducibility. The same ROI was applied to ADC maps for ADC measurement. Each lesion ROI was delineated twice, and the mean value was used for analysis, Interobserver agreement between the two radiologists was evaluated using the intraclass correlation coefficient (ICC).

For each lesion, SyMRI parameters before and after contrast injection were recorded (T_1_-pre, T_2_-pre, PD-pre; T_1_-Gd, T_2_-Gd, PD-Gd), and relative changes (ΔT_1_%, ΔT_2_%, ΔPD%) were calculated as (Gd – Pre)/Pre, Pre- and post-contrast SyMRI images were automatically co-registered by the vendor’s software to minimize misalignment.” When multiple lesions were present, only the largest was analyzed. Discrepancies were resolved by consensus.

An ROI was also drawn in the area of the most prominent enhancement to generate a time–signal intensity curve (TIC) on the GE Advantage Workstation (version 4.7). TICs were classified into three types (persistent, plateau, washout) according to BI-RADS morphology. MRI morphological features—including maximum diameter, lesion shape, margin, enhancement pattern (homogeneous, heterogeneous, rim), and background parenchymal enhancement (BPE; minimal, mild, moderate, marked)—were also evaluated.

Breast fibroglandular tissue (FGT) was visually assessed on pre-contrast images and categorized into three groups: mild (almost entirely fatty + scattered), moderate (heterogeneous), and marked (extreme).

### 1.3 Histopathological evaluation

Clinicopathological data, including ER, PR, Ki-67, and HER2 status, were obtained from the hospital records. ER and PR positivity were defined as nuclear staining in more than 1% of tumor cells. HER2 status was assessed by immunohistochemistry (IHC) using a rabbit monoclonal antibody (clone 4B5, Ventana/Roche) on a Bench Mark ULTRA automated staining platform, following the 2023 ASCO/CAP guidelines. Two experienced pathologists, blinded to imaging results, independently interpreted all slides, and any discrepancies were resolved by consensus. HER2 expression was categorized as follows: 3+ as HER2-overexpression (HER2-oe), 0 as HER2-zero, and 1+ as HER2-low. Equivocal cases (IHC 2+) underwent fluorescence *in situ* hybridization (FISH); cases with HER2 gene amplification were classified as HER2-oe, while non-amplified cases were classified as HER2-low.

### 1.4 Patient grouping

#### 1.4.1 Grouping based on HER2 expression status

- HER2 overexpression group (HER2-oe): IHC 3+ or IHC 2+ with HER2 gene amplification confirmed by FISH.
- HER2 low expression group (HER2-low): IHC 1+ or IHC 2+ without HER2 gene amplification on FISH.
- HER2 zero expression group (HER2-zero): IHC 0 (no HER2 expression).

#### 1.4.2 Grouping based on HER2 positivity

- HER2-positive group: Included all HER2 overexpression cases (IHC 3+ or IHC 2+ with FISH+).
- HER2-negative group: Included both HER2-low and HER2-zero cases.

*Note: HER2 testing and classification were performed in accordance with the American Society of Clinical Oncology/College of American Pathologists (ASCO/CAP) guidelines.*

### 1.5 Statistical analysis

All statistical analyses and figure generation were performed using SPSS version 27.0 (IBM Corp., Armonk, NY, USA), GraphPad Prism version 9.0 (GraphPad Software, San Diego, CA, USA), and R version 4.4.2 (R Foundation for Statistical Computing, Vienna, Austria). A two-sided P value < 0.05 was considered statistically significant. Categorical variables were expressed as frequencies and percentages [n (%)] and compared using the chi-square test or Fisher’s exact test where appropriate. Continuous variables were first assessed for normality using the Kolmogorov–Smirnov test. Normally distributed variables were presented as mean ± standard deviation (SD) and compared using the independent-samples t test, whereas non-normally distributed variables were expressed as median (interquartile range, IQR) and compared using the Mann–Whitney U test.

Variables with statistical significance in univariate analysis were entered into multivariate logistic regression to identify independent predictors. Before modeling, multicollinearity was assessed using the variance inflation factor (VIF), and all variables included in the final model had VIF < 5. Based on the multivariate results, predictive nomogram models were constructed. Model discrimination was evaluated using receiver operating characteristic (ROC) curves and the area under the curve (AUC), along with sensitivity and specificity. Model calibration was assessed using calibration plots with 1000 bootstrap resamples, and bias-corrected AUC, Brier score, calibration slope, and intercept were calculated. To reduce the risk of overfitting, the number of predictors in the final multivariable models was restricted according to the events-per-variable (EPV > 10) criterion. Decision curve analysis (DCA) was conducted to determine the clinical utility of the models by estimating net benefit across a range of threshold probabilities. Interobserver agreement between the two radiologists for quantitative measurements was evaluated using the intraclass correlation coefficient (ICC), with ICC ≥ 0.80 considered excellent.

### 1.6 Ethics statement

This study was approved by the institutional ethics committee (Approval No. 2023-064) and conducted in accordance with the Declaration of Helsinki; informed consent was waived.

## 2 Results

### 2.1 General clinical characteristics

A total of 138 patients with invasive breast cancer (138 lesions) were included. The mean age was 48.4 ± 6.6 years. The mean ages of the HER2-oe (HER2-oe, *n* = 49), HER2-low (*n* = 55), and HER2-zero (*n* = 34) groups were 48.3 ± 11.4, 42.7 ± 9.9, and 45.3 ± 11.3 years, respectively, with no significant intergroup difference (*P* = 0.063). Immunohistochemical markers, including ER (*P* = 0.894), PR (*P* = 0.337), and Ki-67 (*P* = 0.438), also showed no significant differences among the three groups (**Table 2**).

**Table 2:** Comparison of imaging features and clinicopathological factors among HER2 subgroups.

Feature		HER2 (zero)	HER2 (low)	HER2 (over)	*Overall P*	χ²	*P*_1_	*P*_2_	*P*_3_
		(N = 34)	(N = 55)	(N = 49)					
Margin	well-defined	25	29	28	0.511	1.32	0.784	0.592	0.667
	ill-defined	9	26	21					
Shape	oval	13	20	18	0.072	5.32	0.461	0.162	0.298
	irregular	15	18	23					
	lobulated	6	17	8					
Enhancement pattern	homogeneous	16	26	10	0.031	11.72	0.598	0.077	0.037
	heterogeneous	8	16	28					
	rim enhancement	10	13	11					
Margin morphology	spiculated	21	30	22	0.201	3.14	0.785	0.657	0.693
	smooth	13	25	27					
TIC	I (Persistent)	9	10	8	0.003	12.43	0.528	0.341	0.013
	II (plateau)	14	25	9					
	III (washout)	11	20	32					
FGT	minimal	7	7	9	0.63	0.80	0.884	0.948	0.661
	moderate	17	29	26					
	marked	10	19	14					
ER	+	21	37	32	0.89	0.22	0.960	0.836	0.997
	–	13	18	17					
PR	+	21	37	38	0.33	2.17	0.878	0.284	0.343
	–	13	18	11					
Ki-67	≤30%	12	29	25	0.43	1.65	0.179	0.240	0.954
	>30%	22	26	24					

P_1_, Zero vs. Low;P_2_, Zero vs. Over; P_3_, Low vs. Over.

**Figure 2:**
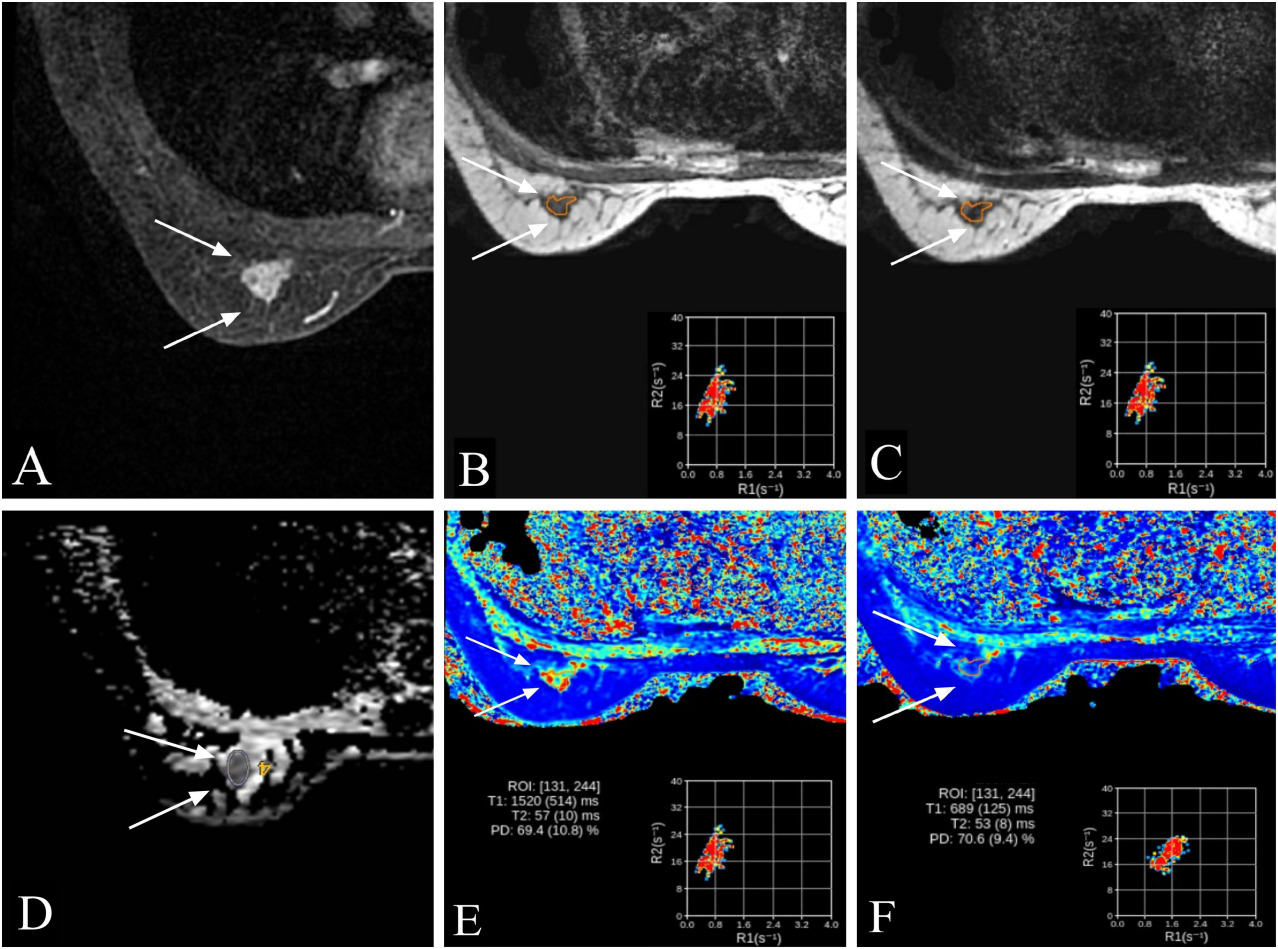
Representative multiparametric MRI findings in a 52-year-old woman with invasive breast cancer and HER2-low expression (IHC 1+). **(A)** DCE-MRI showing a moderately enhancing lesion in the right breast (arrows). **(B, C)** SyMRI parametric maps (T1-pre and T2-pre) with ROI placement. **(D)** ADC map from MUSE-DWI demonstrating restricted diffusion (arrows). **(E)** Pre-contrast SyMRI T1 mapping (T1-pre) with quantitative measurement. **(F)** Post-contrast SyMRI T1 mapping (T1-Gd) showing shortened relaxation time within the lesion.

**Figure 3:**
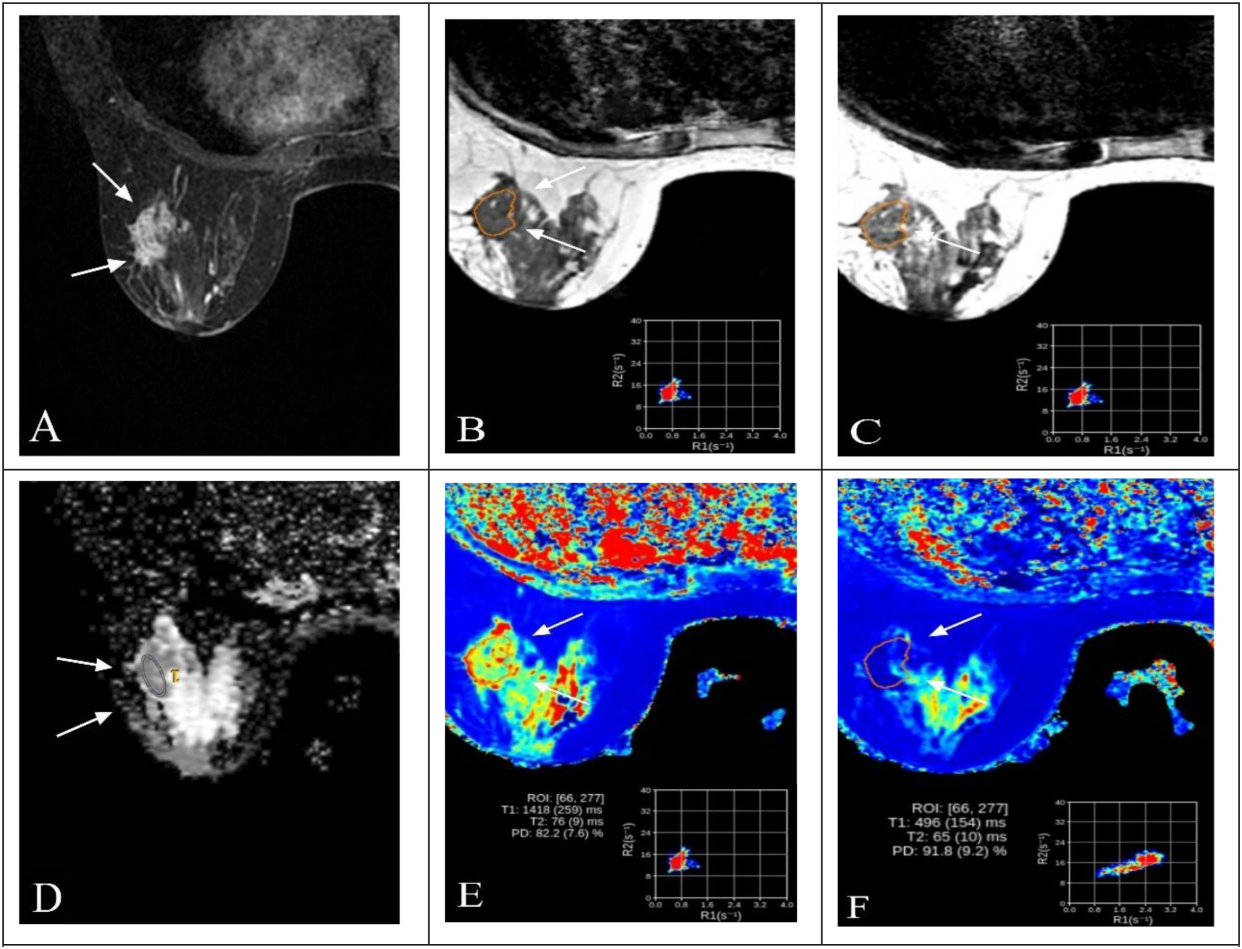
Representative multiparametric MRI findings in a 63-year-old woman with invasive breast cancer and HER2 overexpression (IHC 3+). **(A)** DCE-MRI showing a heterogeneously enhancing lesion in the right breast (arrows). **(B, C)** SyMRI parametric maps (T1-pre and T2-pre) with ROI placement. **(D)** ADC map from MUSE-DWI demonstrating restricted diffusion (arrows). **(E)** Pre-contrast SyMRI T1 mapping (T1-pre) with quantitative measurement. **(F)** Post-contrast SyMRI T1 mapping (T1-Gd) showing shortened relaxation time within the lesion.

Representative multiparametric MRI findings of HER2-low and HER2-overexpressing breast cancers are shown in **Figures 2** and **3**.

### 2.2 Consistency of quantitative parameters

Interobserver reproducibility was excellent for all quantitative parameters: ADC (ICC = 0.876), maximum diameter (ICC = 0.886), T1-pre (ICC = 0.863), T2-pre (ICC = 0.954), PD-pre (ICC = 0.975), T1-Gd (ICC = 0.889), T2-Gd (ICC = 0.934), PD-Gd (ICC = 0.953), T1-Δ % (ICC = 0.877), T2-Δ% (ICC = 0.897), and PD-Δ% (ICC = 0.896), indicating high data stability and reliability.

### 2.3 Imaging morphology and pathological features in relation to HER2 expression

Comparisons of imaging features revealed significant differences in enhancement pattern (*P* = 0.020) and TIC type (*P* = 0.014). Heterogeneous enhancement was more frequent n the HER2-overexpressing (HER2-oe) group than in the HER2-low group (*P* = 0.037) and tended to be higher than in the HER2-zero group (*P* = 0.077). The washout TIC type was more common in the HER2-oe group compared with the HER2-low group (*P* = 0.013), whereas the plateau type was less frequent. Other morphological features, including lesion boundary (*P* = 0.513), shape (*P* = 0.225), margin (*P* = 0.208), and background parenchymal enhancement (*P* = 0.638), showed no significant differences among groups (**Table 2**).

### 2.4 SyMRI quantitative parameters and HER2 expression

Significant intergroup differences were observed for ADC, maximum diameter, T1-pre, T2-pre, T1-Gd, T2-Δ%, and PD-Δ% (*all P* < 0.05), whereas PD-pre, T2-Gd, PD-Gd, and T1-Δ% showed no significant differences (*all P* > 0.05).

Specifically, ADC values were highest in the HER2-oe group, significantly exceeding those in the HER2-zero (*P* = 0.018) and HER2-low groups (*P* = 0.041). Maximum diameter was also greater in the HER2-oe group than in the HER2-zero and HER2-low groups (*all P* < 0.001). T1-pre was lowest in the HER2-low group, significantly lower than in the HER2-zero (*P* = 0.038) and HER2-oe groups (*P* = 0.007). T2-pre was highest in the HER2-oe group, significantly higher than in the HER2-low group (*P* = 0.004). T1-Gd differed among groups (*P* = 0.037), with lower values in the HER2-oe group compared with the HER2-zero (*P* = 0.021) and HER2-low groups (*P* = 0.040). T2-Δ% was higher in the HER2-oe group than in the HER2-zero (*P* = 0.011) and HER2-low groups (*P* = 0.036). In contrast, PD-Δ% was highest in the HER2-zero group, significantly greater than in the HER2-low (*P* = 0.018) and HER2-oe groups (*P* = 0.029) (**Table 3**).

**Table 3:** Comparison of clinical characteristics and multiparametric MRI parameters among breast cancer subgroups with different HER2 expression levels.

Measure	HER2 (zero) (N = 34)	HER2 (low) (N = 55)	HER2(over) (N = 49)	*P*-value	Test statistic	*P*_1_	*P*_2_	*P*_3_
Age (years)	45.31 ± 11.25	42.67 ± 9.89	48.34 ± 11.35	0.063	2.78	0.345	0.479	0.362
ADC value (×10–^3^ mm ^2^/s)	0.921 ± 0.12	0.951 ± 0.13	1.011 ± 0.18	0.032	3.54	0.018	0.041	0.018
Longest diameter (mm)	20.13(17.57,22.57)	22.48(17.93,24.59)	26.28(24.37,27.94)	0.003	11.80^a^	0.713	<0.001	<0.001
T1-pre (ms)	1516 ± 221	1403 ± 243	1361 ± 175	0.024	3.85	0.038	0.007	0.374
T2-pre (ms)	81.8 ± 24.31	76.3 ± 7.63	84.7 ± 13.40	0.014	4.42	0.125	0.427	0.004
PD-pre (pu)	75.24 ± 10.01	83.51 ± 7.67	85.56 ± 7.24	0.404	0.91	0.378	0.871	0.200
T1-Gd (ms)	525.0(460.75,588.47)	521.7(447.95,587.10)	472.1(461.1,598.11)	0.037	3.41^a^	0.089	0.040	0.021
T2-Gd (ms)	68.92 ± 30.12	63.69 ± 8.94	72.78 ± 6.64	0.116	2.19	0.087	0.758	0.082
PD-Gd (pu)	90.55 (85.02, 100.15)	88.1(82.6, 94.2)	86.8(82.8, 95.4)	0.373	1.97^a^	0.116	0.105	0.921
T1-Δ (%)	0.66(0.59,0.67)	0.62(0.57,0.70)	0.62(0.57,0.65)	0.575	0.55^a^	0.997	0.445	0.326
T2-Δ (%)	0.16 ± 0.10	0.14 ± 0.14	0.18 ± 0.15	0.039	3.26	0.929	0.011	0.036
PD-Δ (%)	-0.19 ± 0.25	-0.13 ± 0.18	-0.15 ± 0.16	0.048	3.12	0.018	0.029	0.160

P_1_, Zero vs. Low;P_2_, Zero vs. Over; P_3_, Low vs. Over; a = Kruskal–Wallis H test; Other values = F test.

### 2.5 Predictors of HER2 positivity and nomogram construction

**Figure 4:**
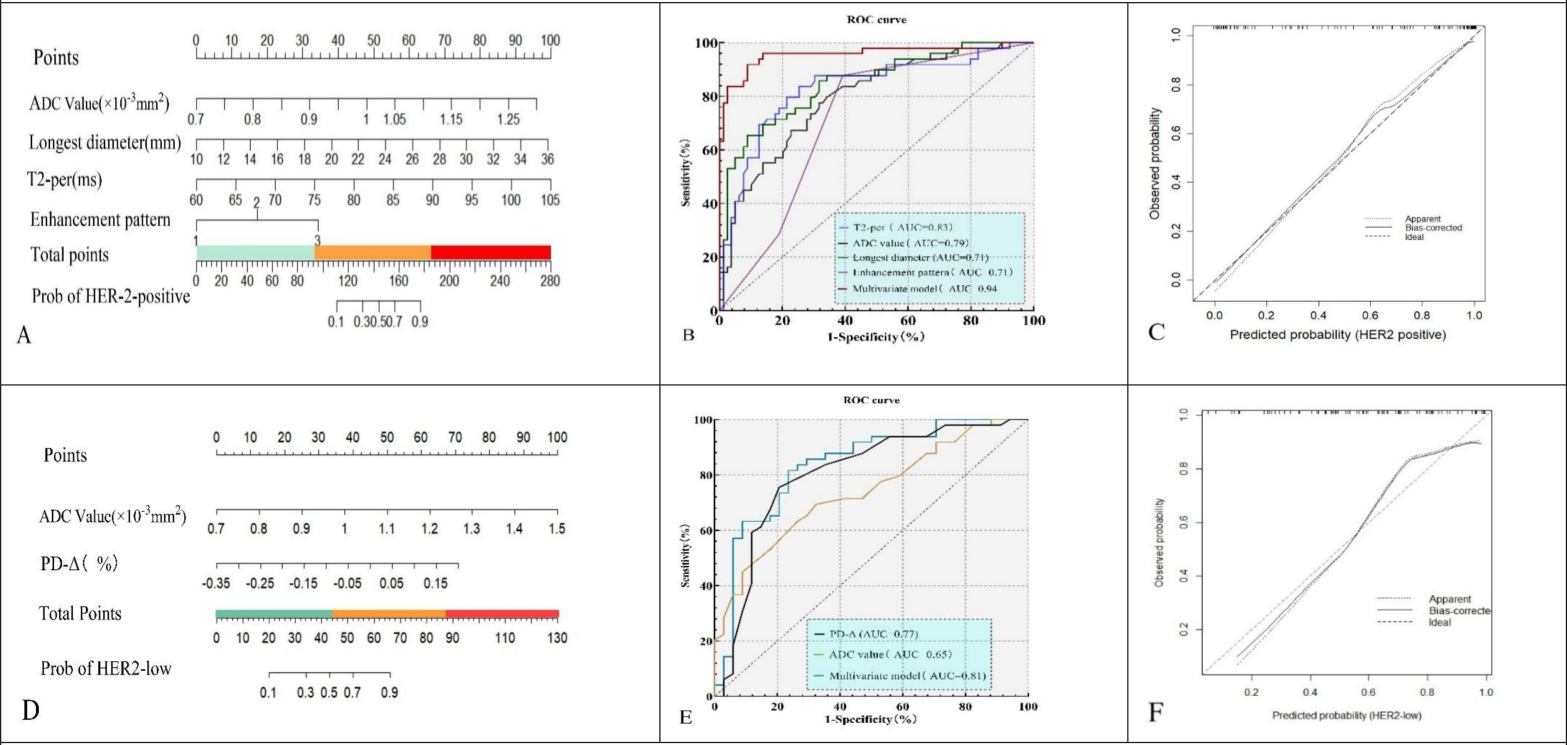
Nomograms **(A, D)**, ROC curves **(B, E)**, and calibration plots **(C, F)** for the HER2-positive model **(A–C)** and HER2-low vs HER2-zero model **(D–F)**.The models yielded AUCs of 0.94 (95% CI: 0.90–0.98) and 0.81 (95% CI: 0.73–0.89), respectively, with good calibration.

Univariate analysis identified ADC, maximum diameter, T2-pre, PD-Δ%, and enhancement pattern as significant predictors of HER2 status (*all P* < 0.05). Multivariate logistic regression confirmed ADC (OR = 6.48, 95% CI: 2.84–14.79, *P* < 0.001), maximum diameter (OR = 1.29, 95% CI: 1.12–1.51, *P* < 0.001), T2-pre (OR = 1.17, 95% CI: 1.07–1.34, *P* < 0.001), and enhancement pattern (type III vs. type I: OR = 1.81, 95% CI: 1.20–2.72, *P* = 0.006) as independent predictors of HER2 positivity. A nomogram was developed based on these variables to predict HER2 positivity (**Table 4**, **Figure 4A**).

**Table 4:** Univariable and multivariable logistic regression analyses of MRI parameters for predicting HER2 positivity in breast cancer.

Variable	Univariable analysis			Multivariable analysis		
	B	*OR* [95% CI, Lower - Upper]	*P*-value	B	*OR* [95% CI, Lower - Upper]	*P*-value
ADC	1.95	7.02 [3.60 - 13.65]	< 0.0001	1.87	6.48 [2.84 - 14.79]	< 0.001
Long diameter	0.293	1.34 [1.12 - 1.51]	< 0.001	0.23	1.29 [1.12 - 1.51]	< 0.001
T1-pre	-0.22	1.01 [0.97 - 1.11]	0.077	–	–	–
T2-pre	0.17	1.18 [1.10 -1.28]	< 0.001	0.16	1.17 [1.07 - 1.34]	< 0.001
T1-Gd	0.11	1.00 [0.97 - 1.01]	0.67	–	–	–
T2-Δ (%)	-1.52	0.21 [0.16 - 2.98]	0.26	–	–	–
PD-Δ (%)	1.23	3.42 [1.73 - 6.76]	< 0.001	–	–	–
Enhancement pattern			< 0.001	–	–	0.002
III vs I	2.24	9.45 [5.06 - 17.56]	< 0.001	0.59	1.81 [1.20 - 2.72]	0.006
II vs I	1.52	4.56 [2.46 - 9.47]	< 0.001	–	–	–
TIC			0.002	–	–	–
III vs I	-0.96	0.39 [0.12 - 1.21]	0.101	–	–	–
II vs I	0.62	1.86 [0.68 - 5.13]	0.124	–	–	–

OR, odds ratio; CI, confidence interval; ADC, apparent diffusion coefficient; TIC, time–intensity curve: type I = persistent, type II = plateau, type III = washout. Enhancement pattern: I=homogeneous, II= heterogeneous, III= rim enhancement.

### 2.6 Predictors of HER2-low status and nomogram construction

Univariate analysis showed ADC, T1-pre, and PD-Δ% were significantly associated with HER2-low status (*all P* < 0.05). Multivariate logistic regression demonstrated ADC (OR = 2.94, 95% CI: 1.17–7.37, *P* = 0.021) and PD-Δ% (OR = 3.86, 95% CI: 1.74–8.58, *P* = 0.001) as independent predictors of HER2-low. A nomogram was constructed to differentiate HER2-low from HER2-zero based on these predictors (**Table 5**, **Figure 4D**).

**Table 5:** Univariable and multivariable logistic regression of MRI parameters for predicting HER2 status in breast cancer.

Variable	Univariable analysis			Multivariable analysis		
	B	*OR* [95% CI, Lower - Upper]	*P-*value	B	*OR* [95% CI, Lower - Upper]	*P-*value
ADC	1.25	3.49 [1.45-8.15]	0.004	1.08	2.94 [1.17-7.37]	0.021
Longest diameter	-0.006	0.94 [0.912-1.084]	0.89	–	–	–
T1-pre	-0.04	0.99 [0.96-1.01]	0.054	–	–	–
T2-pre	-0.02	0.98 [0.93-1.17]	0.22	–	–	–
T1-Gd	0.11	1.12 [0.97-1.21]	0.18	–	–	–
T2-Δ (%)	0.19	1.20 [0.29-3.42]	0.92	–	–	–
PD-Δ (%)	1.23	3.42 [1.73-6.76]	< 0.001	1.35	3.86 [1.74-8.58]	0.001
Enhancement pattern			0.15	–	–	–
I vs III	-1.31	0.27 [0.06-1.09]	0.67	–	–	–
I vs II	-0.67	0.50 [0.11-2.91]	0.38	–	–	–
TIC			0.52	–	–	–
I vs III	0.67	1.95[1.22-1.93]	0.39	/	/	/
I vs II	-0.20	0.82 [0.30-2.39]	0.71	/	/	/

OR, odds ratio; CI, confidence interval; ADC, apparent diffusion coefficient; TIC, time–intensity curve: type I = persistent, type II = plateau, type III = washout. Enhancement pattern: I=homogeneous, II= heterogeneous, III= rim enhancement.

### 2.7 ROC and calibration analyses

#### 2.7.1 HER2-positive group

ROC analysis showed that T2-pre had an AUC of 0.830 (sensitivity 72.6%, specificity 83.6%), ADC an AUC of 0.790 (sensitivity 69.8%, specificity 80.1%), maximum diameter an AUC of 0.710 (sensitivity 65.7%, specificity 78.8%), and enhancement pattern an AUC of 0.710 (sensitivity 77.6%, specificity 67.8%). The combined model achieved an AUC of 0.940 with sensitivity of 81.9% and specificity of 86.3%, significantly outperforming individual predictors (**Figure 4B**). Calibration analysis with bootstrap resampling (*B* = 1000) demonstrated good agreement between the bias-corrected curve and the ideal line, with corrected AUC of 0.930, Brier score of 0.080, calibration slope of 0.910, and intercept of 0.030 (**Figure 4C**).

#### 2.7.2 HER2-low group

For HER2-low, ADC yielded an AUC of 0.650 (sensitivity 78.3%, specificity 83.5%), and PD-Δ% yielded an AUC of 0.770 (sensitivity 82.4%, specificity 79.8%). The combined model achieved an AUC of 0.810, with sensitivity of 79.8% and specificity of 83.6%, superior to the single predictors (**Figure 4E**). Internal validation with bootstrap resampling (*B* = 1000) showed corrected AUC of 0.830, Brier score of 0.182, calibration slope of 1.020, and intercept of –0.030, confirming good agreement between predicted and observed probabilities (**Figure 4F**).

### 2.8 Model calibration and clinical utility (HER2-positive and HER2-low)

**Figure 5:**
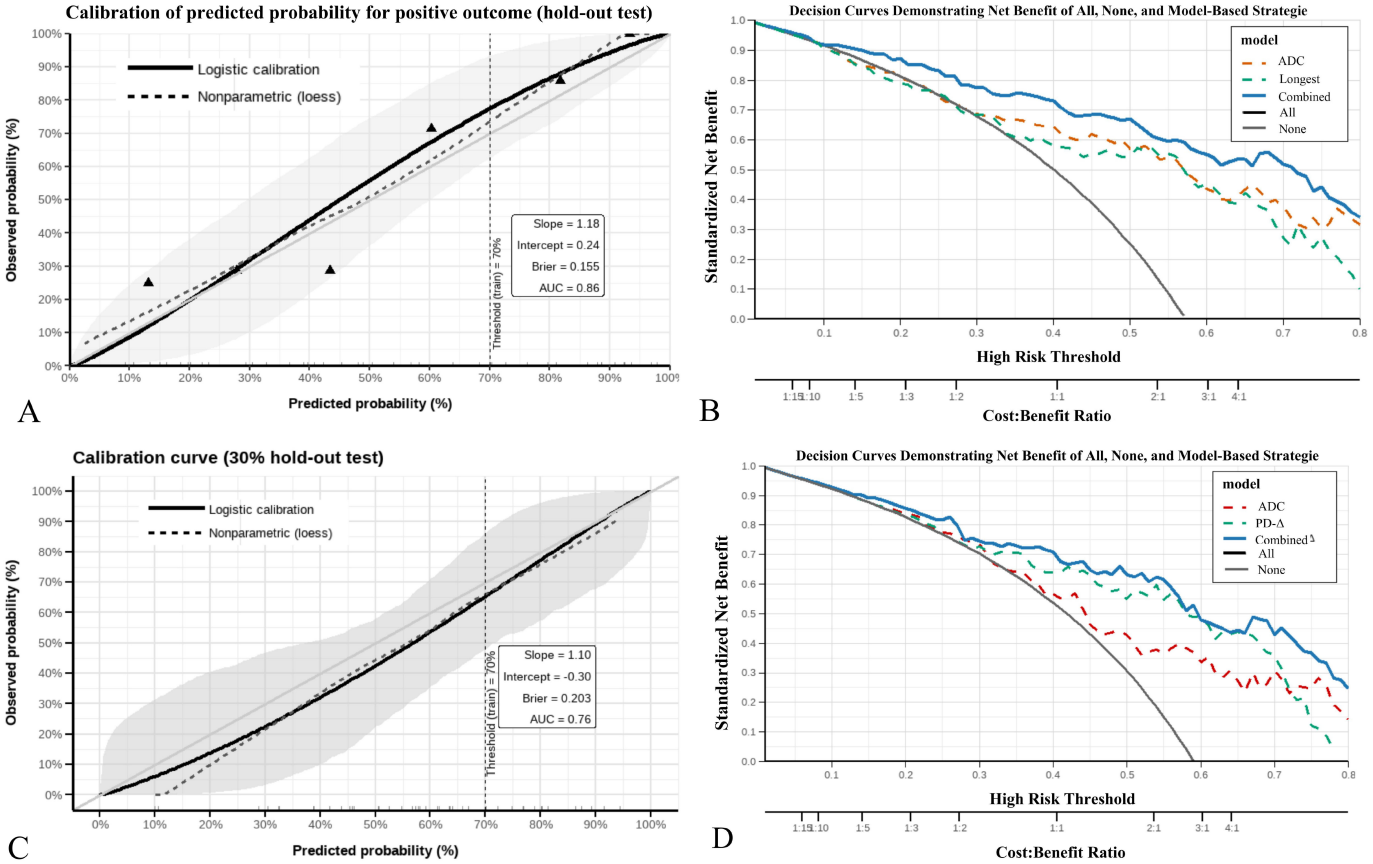
Calibration and decision curve analysis of prediction models. **(A, B)** The HER2-positive model showed good calibration (slope = 1.18, intercept = 0.24) and provided higher net benefit than single predictors or treat-all/none strategies across threshold probabilities of ~0.10–0.75. **(C, D)** The HER2-low model demonstrated acceptable calibration (slope = 1.10, intercept = −0.30) and yielded greater clinical net benefit within thresholds of ~0.20–0.70.

For the HER2-positive model, the calibration curve showed good agreement between predicted and observed probabilities (slope = 1.18, intercept = 0.24, Brier = 0.155) (**Figure 5A**). DCA indicated that the combined model achieved higher net benefit than single predictors and the “treat-all” or “treat-none” strategies across a threshold range of ~0.10–0.75 (**Figure 5B**).

For the HER2-low model, calibration also showed acceptable alignment with the ideal line (slope = 1.10, intercept = –0.30, Brier = 0.203) (**Figure 5C**). The combined model provided greater net clinical benefit than individual parameters and default strategies within thresholds of ~0.20–0.70 (**Figure 5D**).

## 3 Discussion

The molecular heterogeneity of breast cancer contributes to its heterogeneous therapeutic responses and prognostic outcomes. Following the recognition of HER2-low as a clinically actionable subtype, particularly after the DESTINY-Breast04 trial demonstrated that HER2-low patients could benefit from trastuzumab deruxtecan (T-DXd) (5), precise stratification of HER2 expression has become increasingly important. However, most studies still focus on differentiating HER2-positive from HER2-negative disease, while HER2-low versus HER2-zero remains underexplored. In this study, multiparametric MRI combining SyMRI, MUSE-DWI, and DCE-MRI effectively captured HER2-related biological differences and showed strong potential for distinguishing both HER2-positive and HER2-low subtypes.

From a clinicopathological perspective, no significant differences were observed among HER2-zero, HER2-low, and HER2-oe groups with respect to age, ER, PR, or Ki-67. These findings underscore the limited specificity of conventional clinicopathological markers for HER2 stratification. Prior reports have been inconsistent: some demonstrated associations between HER2 positivity and ER/PR negativity or elevated Ki-67, whereas others failed to confirm such correlations (14–16). This further emphasizes that clinicopathological parameters alone are insufficient to resolve the heterogeneity of HER2 expression.

In terms of morphological imaging features, HER2-oe tumors more frequently exhibited heterogeneous enhancement, washout-type TICs, and larger tumor diameters, consistent with their high proliferative activity and abnormal angiogenesis. Previous DCE-MRI and radiomics studies have similarly indicated that such features are associated with poor prognosis (17–19). Our findings validated enhancement pattern and maximum diameter as independent predictors of HER2 positivity. By contrast, no significant differences were observed between HER2-low and HER2-zero, suggesting that morphological characteristics alone have limited discriminative value in stratifying HER2-negative subtypes.

Previous studies have reported inconsistent findings regarding ADC values in HER2-oe tumors: some suggested that increased cellular density in HER2-oe lesions results in reduced ADC values (12), whereas others observed that necrosis, stromal edema, and elevated vascular permeability associated with HER2 overexpression expand the extracellular space, thereby leading to increased ADC values (20, 21). These discrepancies may be attributable to pathological heterogeneity across different stages of tumor progression as well as variations in ROI delineation methods. In addition, conventional SS-EPI DWI is susceptible to geometric distortion and low signal-to-noise ratio, which may further exacerbate inter-study inconsistencies (10).

In this study, MUSE-DWI improved the stability of ADC measurements by reducing artifacts and enhancing spatial resolution. Notably, ADC values were highest in the HER2-overexpressing group and lowest in the HER2-zero group. HER2-oe tumors are more likely to present with necrosis, stromal edema, and increased vascular permeability, all of which expand the extracellular space and elevate water diffusivity, potentially counteracting the reduction expected from increased cellularity (22, 23). Moreover, lesions smaller than 10 mm were excluded, reducing the proportion of small, highly cellular tumors; larger HER2-overexpressing lesions are more prone to necrotic or edematous changes, which may further amplify the influence of necrotic regions on the overall mean ADC value, thereby contributing to higher ADC values. These findings suggest that ADC reflects the complex characteristics of the tumor microenvironment.

Among SyMRI-derived parameters, T2-pre was significantly higher in HER2-oe tumors and remained an independent predictor of HER2 positivity. This may reflect angiogenesis and interstitial fluid accumulation associated with HER2 overexpression, consistent with prior reports of SyMRI-T2 demonstrating diagnostic utility in differentiating benign from malignant breast lesions (24, 25).

In this study, PD-Δ% was found to have value in differentiating HER2-low from HER2-zero tumors, and this difference is more likely attributable to intrinsic variations in tissue structural integrity and microenvironmental stability rather than measurement error. Although classified as HER2-negative, HER2-low tumors retain a minimal level of HER2 expression (26). They tend to preserve epithelial characteristics and cell–cell adhesion, with a relatively dense extracellular matrix and more uniform water distribution, resulting in smaller fluctuations in PD-Δ%. In contrast, HER2-zero tumors completely lack HER2 signaling and exhibit greater structural heterogeneity. Variability in cell junctions, stromal composition, and microvascular perfusion may lead to unstable changes in PD before and after contrast administration, manifesting as higher dispersion of PD-Δ%. This suggests that PD-Δ% reflects tissue structural integrity and microenvironmental stability to some extent, rather than merely contrast agent kinetics. Additionally, the HER2-zero group may include a subset of triple-negative breast cancers, which more frequently exhibit necrosis and stromal edema, potentially contributing to further variability in PD-Δ% and reinforcing intragroup heterogeneity (27).

By comparison, T1-Gd, T2-Gd, T2-Δ%, and T1-pre showed intergroup differences but were not retained in multivariate analysis. Their limited predictive value may be explained by the pharmacokinetics of contrast agents, timing of image acquisition, and insufficient biological specificity (28–30). In this study, SyMRI acquisition was performed 9–10 minutes after contrast injection, a phase when longitudinal relaxation effects are attenuated, potentially reducing T1-Gd stability while rendering T2-Gd differences more pronounced. T2-Δ% is influenced by multiple factors including perfusion, stromal water content, and tissue architecture, leading to limited specificity for HER2 status and reduced predictive performance.

This study has several limitations. First, it was a single-center retrospective study with a relatively small sample size and no external or temporal validation, which may restrict the generalizability of the predictive models. Second, quantitative parameters were extracted from manually drawn 2D ROIs on SyMRI and MUSE-DWI images acquired from a single vendor, which may introduce observer bias and limit reproducibility across different scanners; therefore, multicenter prospective studies with standardized protocols and 3D whole-lesion analyses are needed to further validate our findings (31, 32).

This imaging-based HER2 stratification demonstrated strong diagnostic performance and potential clinical relevance. Calibration and decision curve analyses showed good agreement between predicted and observed probabilities and a meaningful net clinical benefit. Given that HER2-low patients may benefit from antibody–drug conjugates (e.g., T-DXd), a noninvasive approach to identify this subgroup is of substantial clinical value. Our findings suggest that SyMRI combined with MUSE-DWI may help identify potential candidates for ADC therapy. Compared with radiomics or AI-based models, this quantitative MRI approach offers greater biophysical interpretability and reproducibility, providing a transparent and complementary framework for clinical translation. Prospective multicenter validation is still warranted before clinical implementation.

## Data Availability

The original contributions presented in the study are included in the article/**Supplementary Material**. Further inquiries can be directed to the corresponding author.
