# Supplementary figures and images for: Multiparametric quantitative MRI combining SyMRI and MUSE-DWI for noninvasive stratification of HER2 status in breast cancer
Source: Front Oncol. 2025 Dec 5;15:1709170. doi: 10.3389/fonc.2025.1709170 (PMC12714661; doi:10.3389/fonc.2025.1709170)

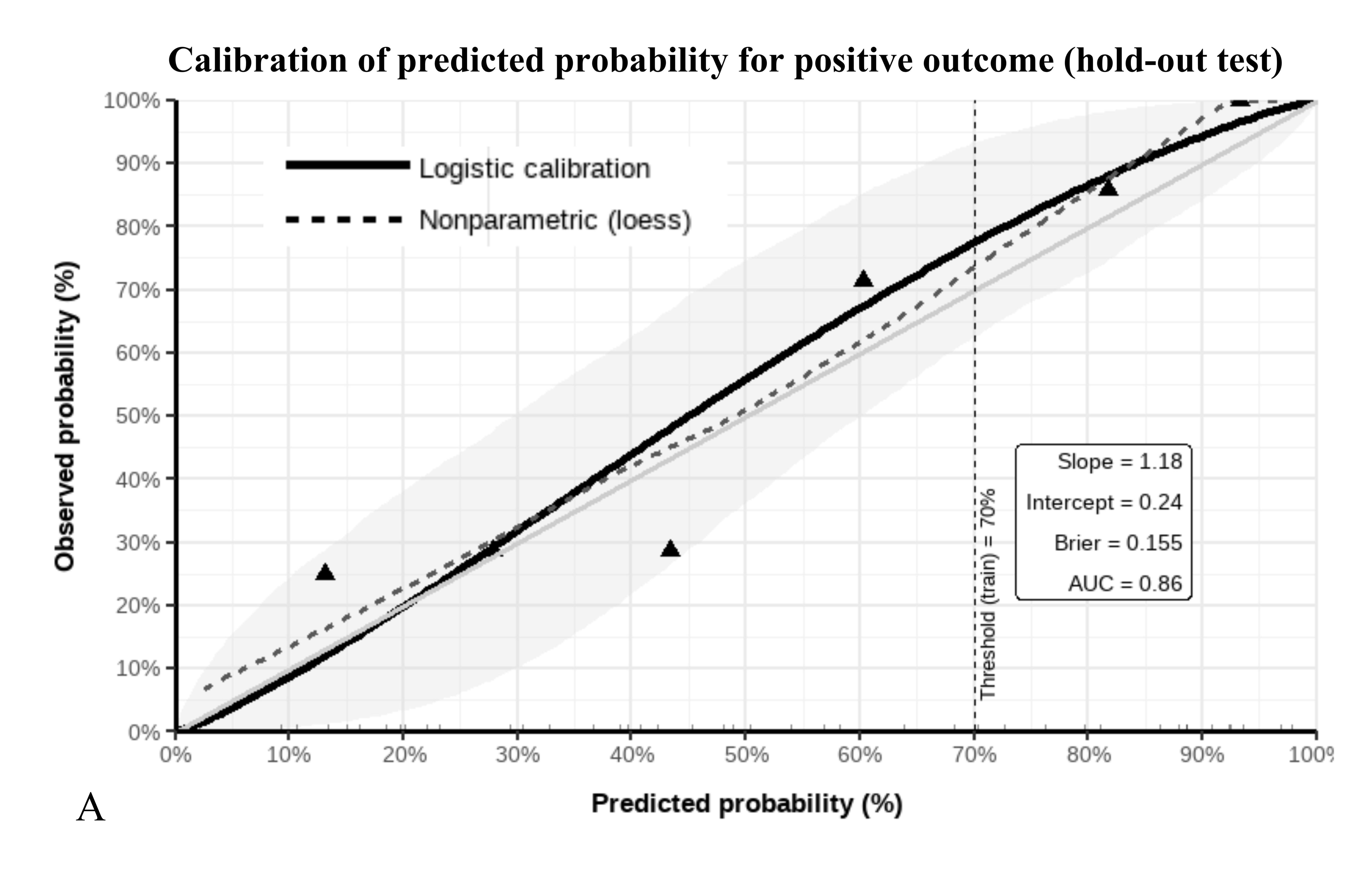

Supplement: Supplementary file 1 [file Image1.jpeg]

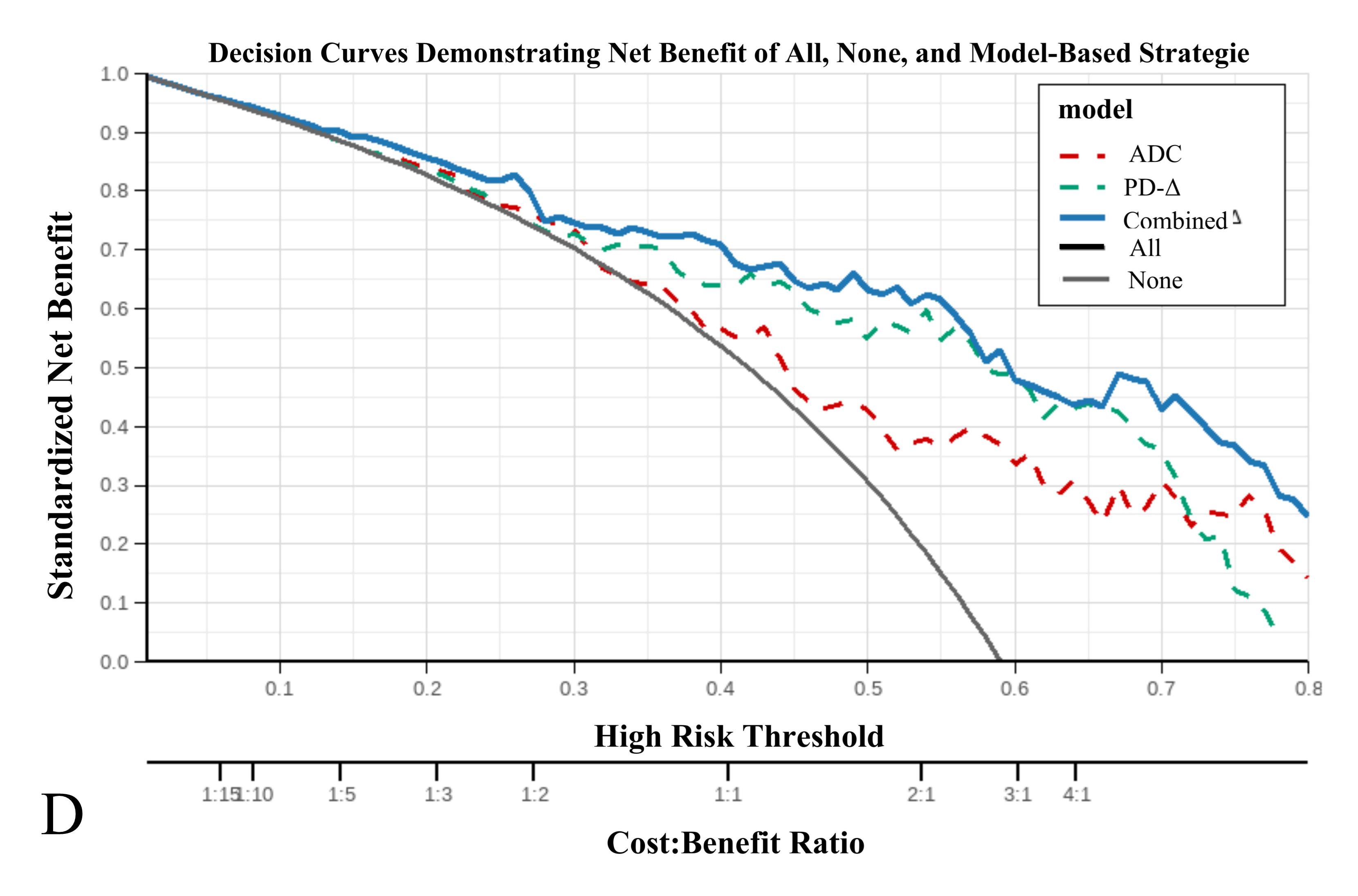

Supplement: Supplementary file 2 [file Image2.jpeg]

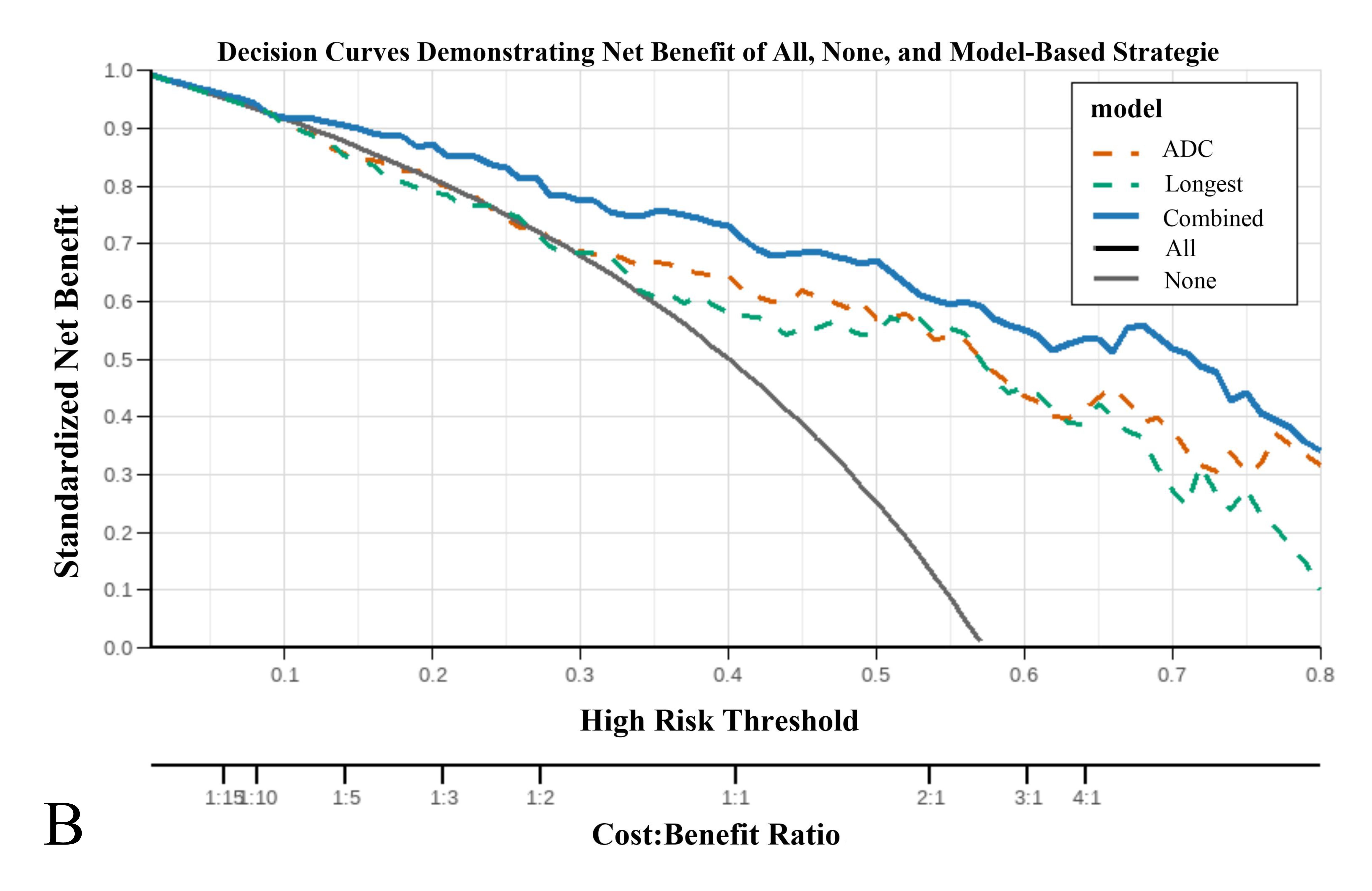

Supplement: Supplementary file 3 [file Image3.jpeg]

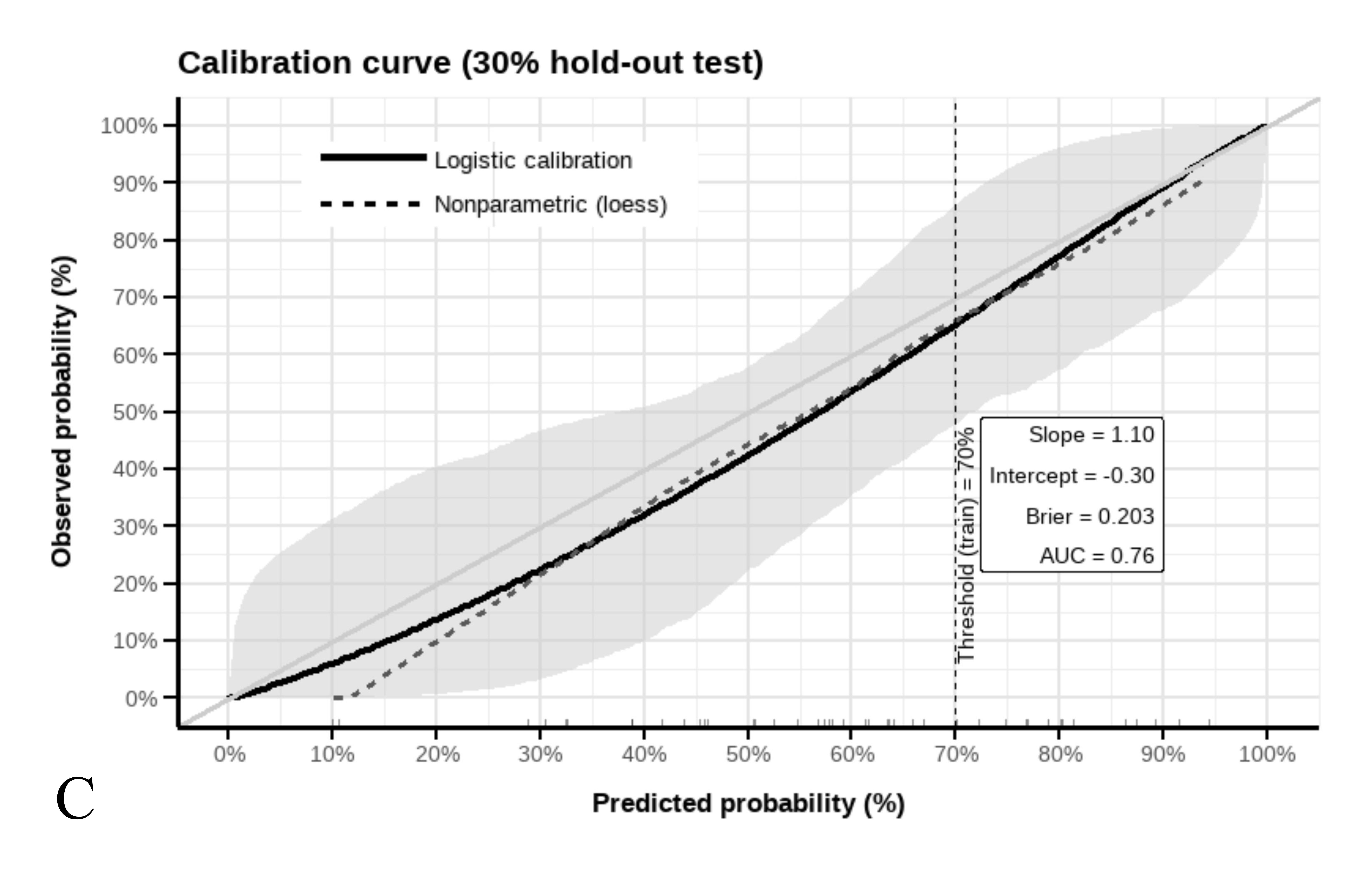

Supplement: Supplementary file 4 [file Image4.jpeg]
